# Supplementary material for: Effect of Moderate to Severe Hepatic Steatosis on Vaccine Immunogenicity against Wild-Type and Mutant Virus and COVID-19 Infection among BNT162b2 Recipients
Source: Vaccines (Basel). 2023 Feb 21;11(3):497. doi: 10.3390/vaccines11030497 (PMC10054100; doi:10.3390/vaccines11030497)
Supplement: Supplementary file 1 [file vaccines-11-00497-s001.zip › vaccines-2188829-supplementary.pdf]

Figure S1. Seroconversion rate of D21, D56, D180 between NAFLD and control groups for different SARS-CoV-2 variants

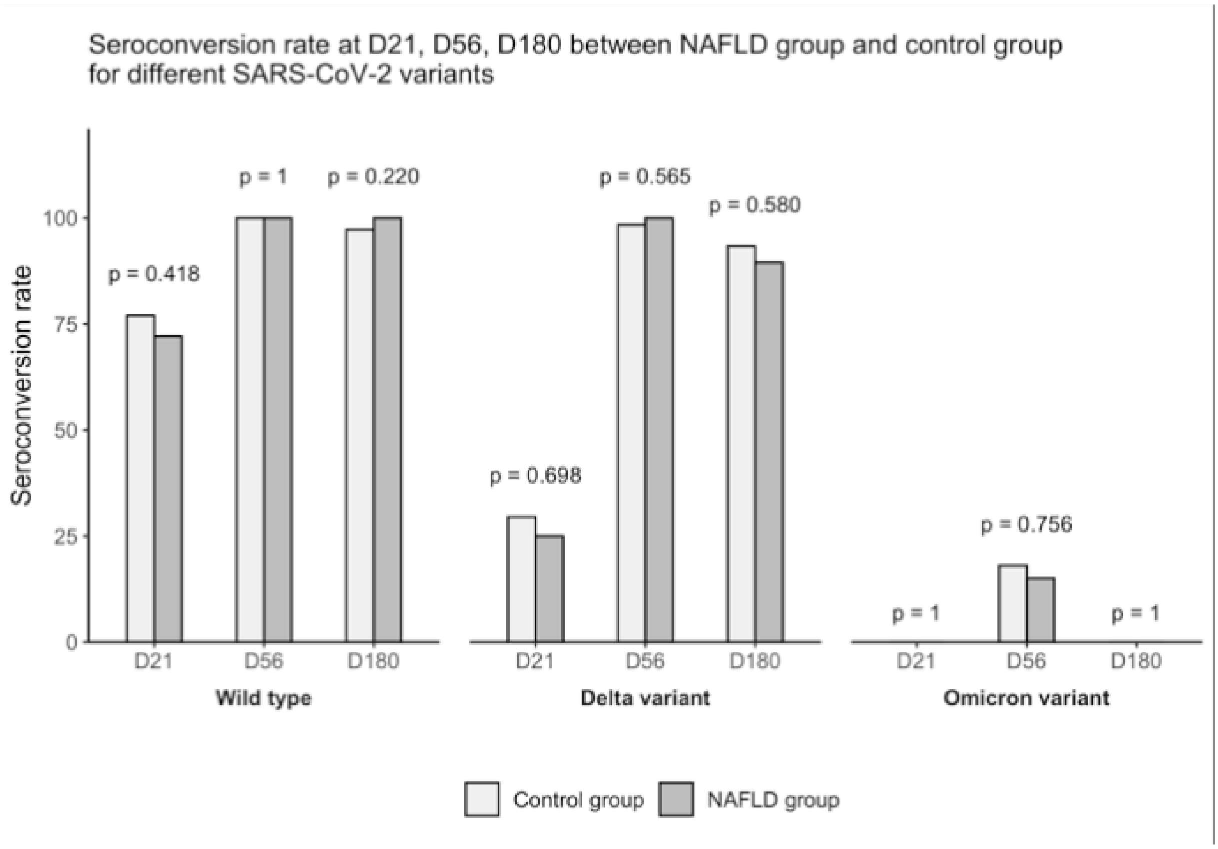

Figure S2. vMN GMT at D21, D56, D180 between NAFLD and control groups for different SARS-CoV-2 variants

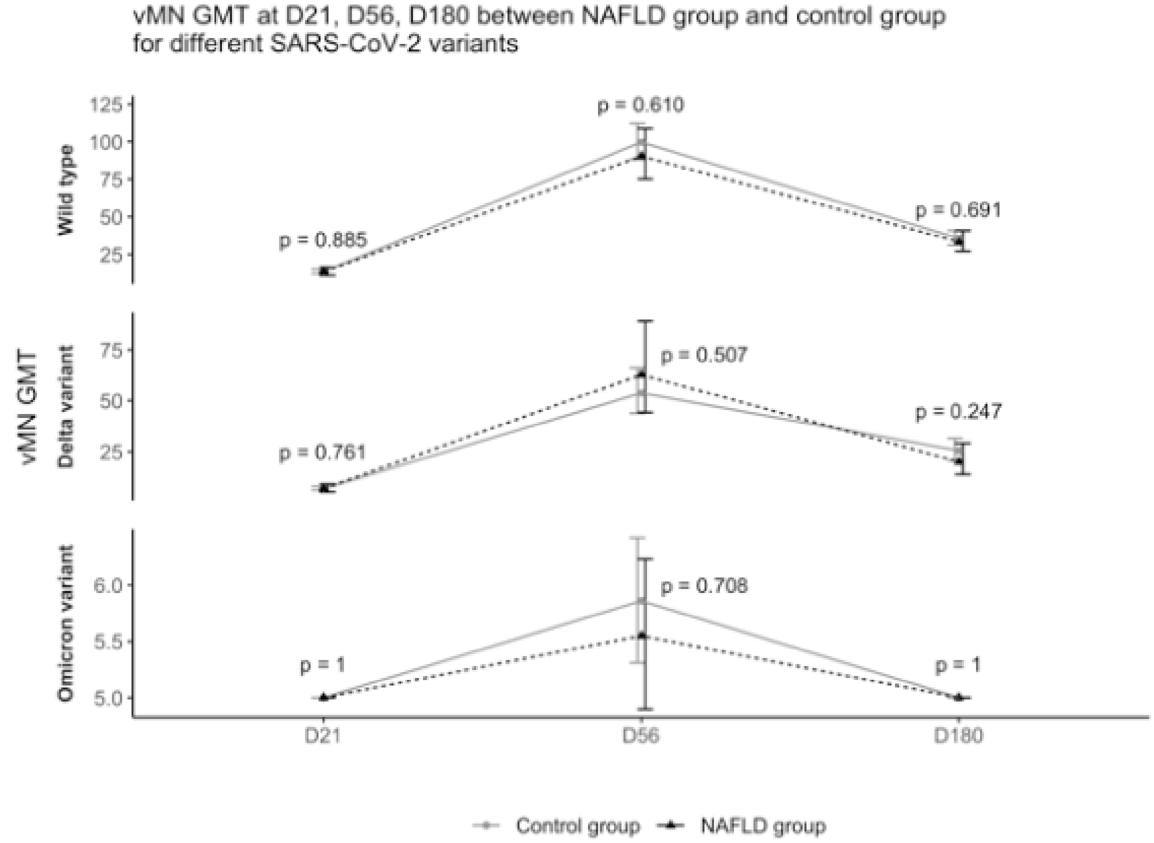

Table S1. Antibody responses to SARS-CoV-2 (wild type, delta variant, omicron variant) among BNT162b2 recipients (sensitivity analysis)

	NAFLD#	Control#	P-value
Wild type			
Seroconversion rate *			
D21	74/99 (74.7)	122/160 (76.3)	0.784
D56	99/99 (100)	158/158 (100)^	1
D180	79/79 (100)^	114/118 (96.6)^	0.098
vMN GMT			
D21	14.29 (12.06-16.95)	13.08 (11.59-14.73)	0.456
D56	96.65 (83.10-112.17)	97.46 (84.78-112.17)	0.704
D180	36.00 (30.57-42.52)	34.33 (29.37-40.04)	0.656
***Delta* variant**			
Seroconversion rate *			
D21	8/32 (25)	15/49 (30.6)	0.584
D56	32/32 (100)	48/49 (98.0)	0.416
D180	29/31 (93.5)^	44/48 (91.7)^	0.758
vMN GMT			

D21	6.77 (5.47-8.33)	7.02 (5.99-8.25)	0.647
D56	60.37 (46.53-78.26)	53.08 (41.68-68.03)	0.614
D180	21.87 (16.78-28.50)	25.56 (19.89-32.79)	0.374
***Omicron* variant**			
Seroconversion rate *			
D21	0/32 (0)	0/49 (0)	1
D56	5/32 (15.6)	9/49 (18.4)	0.750
D180	0/31 (0)^	0/48 (0)^	1
vMN GMT			
D21	UD (UD)	UD (UD)	1
D56	UD (UD)	UD (UD)	1
D180	UD (UD)	UD (UD)	1

Note: Data are displayed as median (interquartile range) and number (%), ^data not available were excluded.
Abbreviations: NAFLD, non-alcoholic fatty liver disease
*Seroconversion rate was considered as positive if MN titre ≥10.
#Sensitivity analysis was performed by reclassifying subjects with mild hepatic steatosis into NAFLD group

Table S2. Comparison of seroconversion rate of neutralizing antibody to SARS-CoV-2 wild type between infected and non-infected subjects*

	Infected (n=55)	Non-infected (n=200)	P-value
D21	42/55 (76.4)	152/200 (76.0)	0.956
D56^	54/54 (100)	199/199 (100)	1
D180#	42/43 (97.7)	148/151 (98.0)	0.890

Note: Data are displayed as number (%), ^data not available were excluded.
* missing data of infection outcome in 4 subjects
^D56: missing data of seroconversion in 2 subjects
D180: missing data of seroconversion in 61 subjects

Table S3. Adverse reactions after either dose of BNT162b2

	All (n=259)	Hepatic steatosis (n = 68)	Control (n = 191)	P-value
Total reactions within 7 days after each injection				
Any	240 (92.7)	63 (92.6)	177 (92.7)	0.343
Grade 3 or above*	0 (0)	0 (0)	0 (0)	--
Injection site adverse reactions	232 (89.6)	59 (86.8)	173 (90.6)	0.412
Pain	230 (88.9)	59 (86.8)	171 (89.5)	0.775
Redness	40 (15.4)	12 (17.6)	28 (14.7)	0.531
Swelling	76 (29.3)	21 (30.9)	55 (28.8)	0.500
Itch	29 (11.2)	8 (11.8)	21 (11.0)	0.593
Systemic adverse reactions	174 (67.2)	52 (76.5)	122 (63.9)	0.231
Fever	48 (18.5)	16 (23.5)	32 (16.8)	0.469
Chills and rigors	33 (12.7)	12 (17.6)	21 (11.0)	**0.042**
Muscle pain			66 (34.6)	**0.075**

	101 (39.0)	35 (51.5)		
Joint pain	39 (15.1)	12 (17.6)	27 (14.1)	**0.026**
Headache	79 (30.5)	24 (35.3)	55 (28.8)	0.639
Fatigue	136 (52.5)	41 (60.3)	95 (49.7)	0.118
Nausea	19 (7.3)	6 (8.8)	13 (6.8)	**0.027**
Vomiting	5 (1.9)	2 (2.9)	3 (1.6)	0.244
Diarrhoea	26 (10.0)	7 (10.3)	19 (9.9)	0.815
Skin rash	11 (4.2)	3 (4.4)	8 (4.2)	0.624
Facial drooping	1 (0.4)	0 (0)	1 (0.5)	0.55

Note: Data are displayed as number (%).
*Grade 3 or above: severe or life threatening reactions
